# Multidisciplinary Approach to Determine the Optimal Time and Period for Extracting the Essential Oil from *Mentha suaveolens* Ehrh †

**DOI:** 10.3390/molecules20069640

**Published:** 2015-05-26

**Authors:** Stefania Garzoli, Adele Pirolli, Elisabetta Vavala, Antonella Di Sotto, Gianni Sartorelli, Mijat Božović, Letizia Angiolella, Gabriela Mazzanti, Federico Pepi, Rino Ragno

**Affiliations:** 1 Department of Drug Chemistry and Technology, “Sapienza” University, P.le Aldo Moro 5, 00185 Rome, Italy; E-Mails: stefania.garzoli@uniroma1.it (S.G.); federico.pepi@uniroma1.it (F.P.); 2 Rome Center for Molecular Design, Department of Drug Chemistry and Technology, “Sapienza” University, P.le Aldo Moro 5, 00185 Rome, Italy; E-Mails: adele.pirolli@uniroma1.it (A.P.); gianni.sartorelli@libero.it (G.S.); mijatboz@gmail.com (M.B.); 3 Department of Public Health and Infectious Diseases, “Sapienza” University, P.le Aldo Moro 5, 00185 Rome, Italy; E-Mails: vavala.elisabetta@libero.it (E.V.); letizia.angiolella@uniroma1.it (L.A.); 4 Department of Physiology and Pharmacology “Vittorio Erspamer”, “Sapienza” University, P.le Aldo Moro 5, 00185 Rome, Italy; E-Mails: antonella.disotto@uniroma1.it (A.D.S.); gabriela.mazzanti@uniroma1.it (G.M.)

**Keywords:** *Mentha suaveolens*, GC/MS, essential oils, piperitenone oxide

## Abstract

A comprehensive study on essential oils (EOs) extracted from some *Mentha suaveolens* L. samples, collected in the countryside of Tarquinia, is reported. In this study, the procedure for essential oil preparation, in terms of harvesting and extraction time, was analyzed in detail for the first time. The GC/MS analysis, carried out on 18 samples, revealed that piperitenone oxide (PO), the main essential oils’ chemical constituent, is primarily responsible for the related antifungal activity. Nevertheless, EOs with lower PO content indicate that other chemicals, such as para-cymenene, may participate in exerting the EOs’ antifungal effect. Furthermore, the bacterial reverse mutation assay highlighted lack of mutagenic effect in all tested samples. Analysis of the results indicated that for higher activity, the essential oils should be produced with 3 h maximum hydrodistillation, regardless of the harvesting time. Differently, the maximum essential oil yield can be obtained in August and the highest piperitenone oxide percentage is obtainable in July.

## 1. Introduction

Antimicrobial properties of essential oils (EOs) are continuously investigated both *in vitro* [1,2] and *in vivo* [3,4] against a wide range of pathogenic bacteria and fungi. Among practical uses, the use of EOs as feed and food additives for the control of gastrointestinal tract pathogens has been suggested [5,6,7]. In clinical settings, EOs’ topical use is the most promising strategy for both skin and mucous membranes [8]. EOs are generally obtained by hydro or steam distillation from various plant sources and, among them, the genus *Mentha* is an important source of basic raw materials [9]. EOs extracted from various plants of mint genus have been extensively investigated for their biological and antimicrobial activities. However, many of them remain to be thoroughly investigated. In particular, the chemical composition of the essential oil extracted from *Mentha suaveolens* Ehrh is highly variable and depends on several factors among which the most important is the easy hybridization of the *Mentha* species [10,11,12], the harvesting time [13,14], the geographic area [14,15,16,17,18,19,20,21,22,23,24,25] and the part of the plant extracted [26]. A bibliographic survey carried out in Scopus search engine (www.scopus.com, accessed 8 October 2014) revealed that 48 individual articles mentioned the words “*Mentha suaveolens* essential oil” (EOMS) although only a 10th actually reported details on the chemical composition. 

As extensively reported by Lawrence and Šarić-Kundalić, *M. suaveolens* can be classified as “spicatae” line on the basis of the inflorescence characters, but for a more detailed classification the characterization of monoterpenes (reflecting three major metabolic pathways) contained in the essential oil is required [14,19].

Recently, we have focused on the extraction of EOs from wild plants of *M. suaveolens*, collected in the Tarquinia countryside [4,27,28,29] highlighting motivating antimicrobial activities, with a potency against *Candida albicans* similar to that of antifungal drugs [4,27]. The chemical composition of the *M. suaveolens* essential oil from Tarquinia (TEOMS) indicates that, in this region, the plant mostly developed carvon metabolic pathways [14], due to the presence of piperitenone oxide (PO) as the major component. Based on this consideration, the antimicrobial activity found for TEOMS was mainly ascribed to this monoterpene, although deeper investigations are required to ascertain the actual PO mechanism of action. Among the different reported EO extraction methods, TEOMS was mainly obtained by hydrodistillation, using a Clevenger-type apparatus for 3–4 h. During our research on TEOMS, it was observed that, either shortening or extending the hydrodistillation time, a different chemical composition of the essential oil could be obtained with some variation in the related biological activity. As a consequence, to obtain the optimal chemical composition of TEOMS, two main questions arise: “When the plant has to be harvested?” and “How long should the hydrodistillation time be?”

Due to weather conditions, difficulties in reaching the *M. suaveolens* field located in the Tarquinia area and the wild growth of the plant, the period to study TEOMS was restricted to the summer season. Concerning the second question, a fractional hydrodistillation protocol was applied to assess the optimal time of extraction. Herein, in line with our previous studies [4,27], we report a comprehensive study on the TEOMS chemical composition and the related antifungal activity. Furthermore, in order to assess TEOMS safety, mutagenicity studies were carried out on some representative samples.

## 2. Results and Discussion

### 2.1. Essential Oil Extraction

Fresh aerial parts from *M. suaveolens* were subjected to hydrodistillation and the oil was collected at various times (1, 2, 3, 6, 12 and 24 h, Table 1 and Supplemental Material Figure S1) on three different days (see Material and Methods Section). The amount of essential oil varied in function of both the extraction day and the separation intervals. In August and September, the essential oil yields (0.19% and 0.18%, respectively) were more than 2.5 times those of July (0.07%, Table 1), nevertheless the oils showed similar profiles at various extraction times and on the different days. The highest amounts were obtained during the first three extraction phases (1 to 3 h) and the last 12 h of extraction (12 to 24 h). Particularly, during the first three hours, about 70%, 54% and 51% of essential oil was extracted in July, August and September, respectively. Considering the classical hydrodistillation procedure, always reported to be over in three hours [15,16,24] from data reported in Table 1, the extraction yield would have been 0.05%, 0.10% and 0.09% for July, August and September, respectively.

**Table 1:** Amount of essential oil (grams) over time and relative yield % calculated on the fresh plant material.

h ^1^	July		August		September	
	EO (g)	Yield %	EO (g)	Yield %	EO (g)	Yield %
**1**	0.73	0.03	1.67	0.07	1.32	0.05
**2**	1.05	0.04	2.14	0.09	2.27	0.09
**3**	1.17	0.05	2.58	0.10	2.35	0.09
**6**	1.28	0.05	3.09	0.12	2.49	0.10
**12**	1.42	0.06	3.63	0.15	2.75	0.11
**24**	1.72	0.07	4.76	0.19	4.59	0.18

^1^ h = extraction hours.

### 2.2. GC/MS EO Analysis

The GC/MS analysis of the 18 TEOMS samples highlighted the presence of 48 different chemical constituents (Supplemental Material Table S2), having different concentrations in the various fractions. In general, the most abundant compound was piperitenone oxide (PO), characteristic for the TEOMS chemotype. Based on the extraction time, PO percentage reached the maximum during the first three hours (**J1h**, **A2h** and **S3h**, Table 2, Table 3 and Table 4, Supplemental Material Figure S2) to diminish during the last three extraction phases (**J6h**, **J12h** and **J24h**) until disappearing in the **J24h** and **A24h** extracts. The component was particularly abundant in July (87%) and August (77%) samples.

**Table 2:** Chemical composition of TEOMS extracted in July. Values are in % weight rounded to the second decimal place.

# ^1^	Name	Sample ^2^					
		J1h	J2h	J3h	J6h	J12h	J24h
**1**	(−)-spathulenol	2.17	8.18	8.99	14.23		18.35
**4**	3-octanol	0.18					
**5**	3-octanol acetate	0.11					
**6**	α-cadinene		0.24				
**7**	α-cadinol		0.50	1.75	9.09	10.69	7.62
**8**	α-cubebene	0.30	0.31		0.09		
**13**	β-bourbonene	0.17					
**22**	borneol	0.12					
**23**	calamenene	0.58	2.09	2.52	3.75	10.63	4.32
**24**	carbitol				0.55		2.36
**25**	β-caryophyllene oxide	5.32	13.67	12.65	14.37	5.27	9.24
**26**	cinerolone	0.17	0.09				7.71
**27**	citronellylacetate				0.63	3.21	5.45
**28**	copaene				0.06		0.76
**29**	cubenol	0.20		1.92	7.46	6.17	4.24
**31**	δ-cadinene				0.12		4.89
**32**	demelverine	0.59	0.51	2.18	9.52	43.46	20.28
**34**	eucarvone	0.08					
**36**	gamma-cadinene						0.86
**37**	*p*-cymen-8-ol	0.12					
**38**	*p*-menth-1-en-8-ol	0.14					
**40**	piperitenone oxide	87.25	70.59	65.56	26.03	14.00	
**41**	tau-cadinol			0.72	1.39		1.37
**42**	tau-muurolol		0.18	1.23	2.14		3.29
**46**	verbenone	1.29	1.15		2.98	6.56	6.43
**47**	veridiflorol	1.21	2.48	2.49	7.59		2.83

^1^ **#** indicate the compound identification number; ^2^ Samples names were obtained by merging the month first letter and extraction time as reported in Table 1.

**Table 3:** Chemical composition of TEOMS extracted in August. Values are in % weight rounded to the second decimal place.

# ^1^	Name	Sample ^2^					
		A1h	A2h	A3h	A6h	A12h	A24h
**1**	(−)-spathulenol	0.79	1.48	2.47	2.12	0.32	
**4**	3-octanol	0.73			0.11	0.07	
**5**	3-octanol acetate	2.08	1.02	0.90			
**7**	α-cadinol				0.78	0.48	0.09
**8**	α-cubebene	5.07	4.36	10.08	3.64	0.45	0.14
**10**	α-pharnesene	5.15	8.01	16.54	9.09	2.56	0.37
**11**	α-phellandrene	0.11					
**12**	α-pinene	1.84					
**14**	*p*-cymenene	0.87	0.33	2.54	7.74	18.24	35.22
**15**	β-elemene				0.38	0.07	
**16**	β-myrcene	1.42					
**17**	β-ocymene	0.59					
**18**	β-pharnesene	0.73	1.03	2.29	2.24	0.89	0.18
**19**	β-phellandrene	0.42					
**20**	β-pinene	0.68					
**21**	bicyclosesquiphellandrene	0.88	0.98	2.37	1.89	0.41	
**22**	borneol	0.28	0.29	0.39	0.37	0.30	0.20
**23**	calamenene	0.44	1.07	1.56	1.33	0.65	
**25**	β-caryophyllene oxide	0.50	0.32	1.04	8.30	17.25	12.93
**26**	cinerolone				23.12	34.49	38.79
**28**	copaene				0.79	0.47	
**29**	cubenol				0.36	0.09	0.04
**30**	d-limonene	6.22					
**31**	δ-cadinene	0.09	0.27	1.07	2.32	1.54	0.44
**32**	demelverine	0.13	1.10	3.14	7.46	8.82	5.90
**33**	eucalyptol	4.21	0.47				
**36**	δ-cadinene				0.51	0.26	0.06
**37**	*p*-cymen-8-ol	0.21	0.54	0.66	0.94	0.97	0.55
**39**	*p*-menthone	0.39	0.46	2.63	5.70	5.61	2.25
**40**	piperitenone oxide	65.05	77.51	50.01	16.90	2.43	
**44**	thymol				0.39	0.32	0.24
**46**	verbenone	0.27	0.31	0.81	2.47	3.14	2.58
**47**	veridiflorol				0.39	0.17	
**48**	ylangene	0.85	0.43	1.50	0.64		

^1^ **#** indicate the compound identification number; ^2^ Samples names were obtained by merging the month first letter and extraction time as reported in Table 1.

**Table 4:** Chemical composition of TEOMS extracted in September. Values are in % weight rounded to the second decimal place.

# ^1^	Name	Sample ^2^					
		S1h	S2h	S3h	S6h	S12h	S24h
**1**	(−)-spathulenol	3.67	8.95	2.54	3.62	1.69	0.99
**2**	(*z*)-jasmone						0.54
**3**	2-caren-10-al					0.32	0.76
**4**	3-octanol		3.13	4.74	0.82	0.61	1.19
**5**	3-octanol acetate		4.90	2.20	0.15	0.12	0.15
**7**	α-cadinol	0.34		0.49	1.73	1.22	0.44
**8**	α-cubebene			0.23	1.97	0.96	1.70
**9**	α-muurolene	0.09		0.13	0.51	0.58	0.15
**10**	α-pharnesene	1.60	10.15	0.11	12.09	6.42	6.31
**12**	α-pinene						0.83
**14**	*p*-cymenene		0.83		1.09	5.04	26.64
**16**	β-myrcene						0.35
**17**	β-ocymene						0.25
**18**	β-pharnesene	0.30	1.32	0.11	5.46	3.23	0.48
**19**	β-phellandrene						0.10
**21**	bicyclosesquiphellandrene			0.19	2.33	1.16	0.77
**22**	borneol				0.23	0.34	0.14
**23**	calamenene	1.68	3.01	1.26	1.87	1.21	0.80
**25**	β-caryophyllene oxide	14.16	14.08	6.30	5.77	9.65	6.37
**26**	cinerolone	18.82			2.66	13.04	18.96
**28**	copaene	0.18	0.21	0.31	1.55	1.67	0.49
**29**	cubenol	0.40	1.77	0.70	1.44	0.84	0.18
**30**	d-limonene		0.69				4.84
**31**	delta-cadinene	0.89		0.14	3.05	3.33	1.25
**32**	demelverine	6.23	0.68	1.84	2.46	4.90	5.22
**33**	eucalyptol		1.16	0.43			2.24
**35**	eugenol	1.42			2.07	3.16	0.55
**37**	*p*-cymen-8-ol	1.67	9.22	0.37	26.77	24.68	4.52
**38**	*p*-menth-1-en-8-ol		0.55	0.20	0.88	0.72	0.22
**39**	*p*-menthone			4.46	1.21	1.57	3.61
**40**	piperitenone oxide	38.69	35.64	69.52	13.20	5.53	5.61
**43**	terpinen-4-ol			0.16	0.30		0.16
**44**	thymol	1.34			0.25	1.04	0.15
**45**	trans-2-caren-4-ol				0.20		
**46**	verbenone	8.11	1.98	2.30	3.68	5.48	2.28
**47**	veridiflorol	0.42	1.74	0.99	1.80	0.96	0.28
**48**	ylangene				0.83	0.31	0.46

^1^ **#** indicate the compound identification number; ^2^ Samples names were obtained by merging the month first letter and extraction time as reported in Table 1.

Other constituents are characteristic of the extraction time and become important only starting from or after the fourth fractions (6 h). In particularly, in July, demelverine (**32**), present in all samples, showed percentages of about 0.5% to 2.0% in the first three hours (**J1h**–**J3h**) of extraction to rapidly grow up to more than 43% at 12 h extraction (**J12h)**. The same compound in August increased from about 0.1% (**A1h**) to only 8.8% (**A12h**). Still in August, *p*-cymenene and cinerolone were found as characterizing compounds for the last fractions (**A24h**). The first one was always contained in all the samples, increasing from about 0.87% (**A1h**) to a maximum of 35.22% **(A24h**). Cinerolone was absent in the samples collected during the first three hours of extraction (**A1h**–**A3h**),while showing high percentage in the next three phases, reaching the maximum amount of about 38.8% after 24 h of extraction (**A24h**). A third different composition scenario was found in September, although piperitenone oxide was still the most abundant compound, with other randomly distributed components. Medium-high percentages of β-caryophyllene oxide (14.16%) and cinerolone (18.82%) were found after the first hour of extraction (**S1h**). After the 2h extraction (**S2h**), β-caryophyllene oxide decreased while cinerolone disappeared. This last compound appeared again after five hours (**S6h**) up to the last extraction (**S24h**), with a percentage varying from 2.66% to 18.96%. Other important chemical components, including α-farnesene (about 12% in **S6h**), *p*-cymenene (more than 26% in **S24h**) and *p*-cymen-8-ol (about 25% in the **S6h** and **S12h** extractions) were also found. Notably, the complexity of the OE chemical composition increased from July to September, being sample **S24h**—with 36 different chemical components out of the 48—the most complex one (Table 4 and Supplemental Material Figure S3). GC analyses were carried out twice showing reproducible results, nevertheless, no statistical analyses were performed. For comparison purposes, Table 2, Table 3 and Table 4 are also reported as united tables in Supplemental Material Table S3.

### 2.3. Antimicrobial Assay 

The *in vitro* antifungal activity of TEOMS extracted at different times and in different summer months, against the reference strain *C. albicans* ATCC 10231, is reported in Table 5. The results are representative of two independent experiments arranged in triplicate. With few exceptions, the MIC of this strain ranged from 0.1 mg∙mL^−1^, to 0.02 mg∙mL^−1^ for TEOMS extracted in July and August, and 0.1 to 0.2 mg∙mL^−1^ for TEOMS extracted in September, and, in all cases, the extracts showing an interesting antifungal activity were in the first three hours of extraction. For the TEOMS extracted from six to 24 h, the MIC values ranged from 6.25 to 12.5 mg∙mL^−1^. TEOMS extracted in the first 3 h showed good antifungal activities, and in one particular case, extract **A2h** (Supplemental Material Figure S2 and Table 3), the anticandida activity was comparable to that of miconazole (Table 5 and Supplemental Material Table S1) [30], a well-known marketed antifungal synthetic drug. Analysis of results in Table 3, Table 4, Table 5 and Table 6 clearly indicates that PO is very likely the chemical components responsible for the antifungal activities reported in Table 6. In fact, the most active mixtures are those containing higher PO percentages. Decreasing of the PO% led to lower active TEOMS except for the most complex mixture **S24h** where the antifungal activity is only about two times lower than **J1h** (highest PO%) and about seven times lower than **A2h**, the highest active TEOMS. Inspecting PO%, it is clear that it is not the only important constituent, and likely other minor chemicals are endowed with microbiological activities and may co-participate in the inhibition process with some synergistic mechanism similar to that reported recently by us [31,32]. The **S24h** (Table 4 and Supplemental Material Figure S3) chemical complexity could explain the low micromolar antifungal activity (Supplemental Material Table S1) and is somehow in agreement with the phytocomplex hypothesis reported in many other experimental observations [33,34]. Further analysis of data in Table 6 indicates the lack of any correlation between the antimicrobial activity and the cultivation stage of the plant, thus supporting the previous reported data that PO is mainly responsible for the antimicrobial activity. Somehow, this is in contrast with some bibliographic data reporting that EOs obtained during flowering season of a plant exhibit the most significant antimicrobial activity [26,35,36].

**Table 5:** Anti-*Candida albicans* activities of the 18 TEOMS extracts.

Sample ^1^	MIC mg∙mL^−1^	PO %
**J1h**	0.10	87.25
**J2h**	0.10	70.59
**J3h**	0.10	65.56
**J6h**	6.25	26.03
**J12h**	6.25	14.78
**J24h**	12.50	-
**A1h**	0.10	65.05
**A2h**	0.02	77.51
**A3h**	0.10	50.01
**A6h**	0.78	16.90
**A12h**	3.12	2.43
**A24h**	6.25	-
**S1h**	0.20	38.69
**S2h**	0.20	35.64
**S3h**	0.10	69.52
**S6h**	6.25	13.20
**S12h**	6.25	5.53
**S24h**	0.20	5.61
**Miconazole**	0.016	

^1^ Samples names were obtained by merging the month first letter and extraction time as reported in Table 1.

### 2.4. Mutagenicity Test

The mutagenicity assays were carried out on three TEOMS samples, namely **J1h**, **A1h** and **S1h**, being the most abundant and showing the strongest antimicrobial activities against *Candida albicans*. The three samples were firstly assayed for their cytotoxicity in the strains tested and were found to be not cytotoxic up to 400 μg∙plate^−1^ (**J1h**), 50 μg∙plate^−1^ (**A1h**) and 100 μg∙plate^−1^ (**S1h**), respectively (Table 6). The presence of the metabolic activator S9 seemed to reduce the cytotoxicity of all three samples (Table 6). The cytotoxicity profile could be due to the different compositions of the three samples. In particular, **J1h** appeared to be the less toxic one; in contrast, **A1h** exerted the highest cytotoxicity in TA100.

Based on these results, we carried out the mutagenicity test using concentrations lower than that found in the preliminary test (viz. **J1h**, 10 to 350 for all strains; **A1h** and **S1h**, 10 to 150 μg∙plate^−1^ for TA98 and WP2*uvr*A, and 10 to 50 μg∙plate^−1^ for TA100). In the absence of the metabolic activation system, the samples of TEOMS did not produce any increase in the number of revertant colonies for *S. typhimurium* TA98 and TA100 and in *E. coli* WP2*uvr*A, so resulting in them not being mutagenic. Likewise, no mutagenic effects were observed in the presence of S9; these results allow hypothesizing that no genotoxic derivatives were produced from the CYP450-mediated biotransformation of the TEOMS components (Table 8). Conversely, the reference mutagens, 2NF, 2AA, SA and MMS increased the number of revertant colonies about four to eight times with respect to the vehicle control, showing the suitability of the system to detect a genotoxic effect (Table 7).

Present results highlight that the three samples of TEOMS, carrying a different phytochemical composition, exhibited a similar marked antimicrobial activity without inducing mutagenic effects. Overall, the phytocomplex contained in the TEOMS samples appears not to be mutagenic, although the toxicity of each component is not always investigated. In this context, it should be underlined the presence of some compounds, including β-caryophyllene oxide, d-limonene and eucalyptol, known to possess genoprotective properties [37,38,39,40]. It could be argued that for sample A1h (Table 6) the number of revertants could be too low. Nevertheless, the number of revertants “0.3 ± 0.2 **^t^” for sample A1h, at the concentration of 850 μg∙plate^−1^ makes sense: its low value stems from the fact that, at this concentration, A1h is toxic, as indicated by the symbol “t”. The toxicity causes a reduction of the number of revertant colonies with respect to the negative control (vehicle DMSO, Table 6). For the same reason, in the mutagenicity test (Table 7), the higher concentration used of 150 µg∙plate^−l^ did not show cytotoxicity and did not reduce the number of revertant colonies.

**Table 6:** Cytotoxicity of **J1h**, **A1h**, and **S1h** samples from *Mentha suaveolens* essential oil on *Salmonella ser. typhimurium* TA98 and TA100 and of *Escherichia coli* WP2*uvr*A strains, in the absence and presence of the metabolic activator S9 (means ± SEM; *n* = 9 plates).

Sample	Compound Amount (μg∙plate^−1^)	Number of Revertant Colonies					
		TA98		TA100		WP2*uvr*A	
		−S9	+S9	−S9	+S9	−S9	+S9
**J1h**	50	44.0 ± 1.1	59.3 ± 1.8	153.3 ± 10.9	162.3 ± 2.6	48.2 ± 3.4	50.0 ± 2.5
	100	45.2 ± 2.1	53.7 ± 3.5	162.8 ± 10.2	149.2 ± 7.1	42.0 ±6.8	67.0 ± 3.7
	250	43.7 ± 3.3	58.8 ± 3.7	146.7 ± 8.3	152.0 ± 8.3	38.3 ± 1.8	58.8 ± 11.9
	400	38.0 ± 3.0	62.7 ± 6.3	136.0 ± 8.3	149.3 ± 3.2	44.2 ± 2.8	63.7 ± 3.7
	550	29.7 ± 3.4 **^,t^	56.5 ± 6.7	73.7 ± 11.2 **^,t^	129.7 ± 5.4	32.5 ± 5.0 ^~t^	57.2 ± 6.8
	850	15.0 ± 1.1 **^,t^	30.3 ± 2.8 **^,t^	40.0 ± 10.6 **^,t^	97.0 ± 2.3 **^,t^	19.3 ± 3.7 **^,t^	34.7 ± 6.7 **^,t^
**A1h**	50	49.3 ± 3.8	68.0 ± 7.9	144.0 ± 14.0	133.3 ± 23.6	42.7 ± 3.5	77.3 ± 5.8
	100	41.3 ± 3.5	60.7 ± 7.3	90.7 ± 14.3 **^,t^	127.3 ± 12.6	43.1 ± 4.9	74.7 ± 13.2
	250	41.3 ± 3.5 **^,t^	60.0 ± 7.3	73.3 ± 32.8 **^,t^	124.0 ± 16.1 ^~t^	32.0 ± 4.8 **^,t^	77.3 ± 1.3
	400	32.7 ± 4.3 **^,t^	30.4 ± 4.5 **^,t^	79.3 ± 7.4 **^,t^	106.7 ± 3.1 **^,t^	25.3 ± 3.3 **^,t^	57.3 ± 11.9 **^,t^
	550	25.3 ± 3.4 **^,t^	28.7 ± 9.4 **^,t^	64.0 ± 16.5 **^,t^	104.7 ± 8.3 **^,t^	30.7 ± 3.4 **^,t^	28.7 ± 2.8 **^,t^
	850	0.3 ± 0.2 **^,t^	0.5 ± 0.1 **^,t^	11.3 ± 5.2 **^,t^	111.3 ± 8.7 **^,t^	2.1 ± 0.1 **^,t^	12.1 ± 0.5 **^,t^
**S1h**	50	53.3 ± 1.3	54.7 ± 8.1	153.3 ± 4.8	177.3 ± 7.4	59.3 ± 5.9	58.2 ± 3.4
	100	54.0 ± 7.5	50.2 ± 6.7	153.3 ± 9.8	173.3 ± 13.3	50.7 ± 4.3	58.0 ± 5.0
	250	40.0 ± 2.3 **^,t^	61.3 ± 7.1	139.3 ± 9.9 **^,t^	170.7 ± 9.3	49.3±6.1 ^~t^	54.2 ± 2.8
	400	53.3 ± 3.5 **^,t^	56.7 ± 4.8 **^,t^	138.7 ± 6.4 **^,t^	146.0 ± 13.6 ^~t^	46.0 ± 5.2 ^~t^	52.0 ± 3.1 ^~t^
	550	17.3 ± 7.4 **^,t^	38.7 ± 9.3 **^,t^	117.3 ± 8.6 **^,t^	150.7 ± 10.7 **^,t^	44.7 ± 8.5 **^,t^	38.0 ± 2.3 **^,t^
	850	5.1 ± 0.3 **^,t^	11.8 ± 0.6 **^,t^	37.3 ± 12.5 **^,t^	49.3 ± 13.8 **^,t^	22.1 ± 1.3 **^,t^	17.8 ± 0.9 **^,^^t^
**Vehicle**		43.3 ± 2.8 ^a^	49.3 ± 2.1 ^a^	143.2 ± 13.6 ^a^	153.6 ± 17.1 ^a^	45.9 ± 5.2 ^a^	59.7 ± 5.3 ^a^
**Mutagen**		128.1 ± 8.1 ^b^	186.1 ± 15.1 ^c^	608.5 ± 14.9 ^d^	379.2 ± 23.5 ^c^	287.4 ± 17.2 ^e^	323.7 ± 20.4 ^f^

^t^ Toxicity (evaluated as >70% reduction of the number of revertant colonies *vs.* vehicle); ^~t^ Minor toxicity (evaluated as a change in the auxotrophic background growth *vs.* vehicle). ^a^ DMSO 50 µL; ^b^ 2-Nitrofluorene (2 μg∙plate^−1^); ^c^ 2-Aminoanthracene (1 μg∙plate^−1^); ^d^ Sodium azide (1 μg∙plate^−1^); ^e^ Methyl methane sulfonate (500 μg∙plate^−1^); ^f^ 2-Aminoanthracene (10 μg∙plate^−1^); ** *p* < 0.01 *vs.* vehicle (Anova + Dunnett’s multiple comparison post-test).

**Table 7:** Effect of **J1h**, **A1h**, and **S1h** samples from *Mentha suaveolens* essential oil on the number of spontaneous revertant colonies of *Salmonella ser. typhimurium* TA98 and TA100 and of *Escherichia coli* WP2*uvr*A strains, in the absence and presence of the metabolic activator S9 (means ± SEM; *n* = 9 plates).

Sample	Compound Amount (μg∙plate^−1^)	Number of Revertant Colonies					
		TA98		TA100		WP2*uvr*A	
		−S9	+S9	−S9	+S9	−S9	+S9
**J1h**	10	46.0 ± 2.3	59.0 ± 3.2	133.3 ± 14.8	150.5 ± 15.4	49.3 ± 7.4	60.0 ± 2.3
	50	44.0 ± 1.1	59.3 ± 1.8	146.7 ± 8.3	129.7 ± 5.4	48.2 ± 3.4	50.0 ± 2.5
	100	38.0 ± 3.0	53.7 ± 3.5	162.8 ± 10.2	149.2 ± 7.1	39.5 ± 5.0	67.0 ± 3.7
	150	40.0 ± 1.8	55.7 ± 6.4	163.3 ± 11.8	155.3 ± 2.4	38.0 ± 5.0	60.2 ± 5.4
	250	43.7 ± 3.3	58.8 ± 3.7	136.0 ± 8.3	152.0 ± 8.3	38.3 ± 1.8	58.8 ± 11.9
	350	45.2 ± 2.1	62.7 ± 6.3	153.3 ± 10.9	149.3 ± 3.2	44.2 ± 2.8	63.7 ± 3.7
**A1h**	10 (10)	55.1 ± 6.8	48.9 ± 5.2	134.4 ± 13.2	140.8 ± 19.7	52.0 ± 5.4	65.8 ± 7.2
	50 (18)	49.3 ± 3.8	68.0 ± 7.9	144.0 ± 14.0	133.3 ± 23.6	42.7 ± 3.5	77.3 ± 5.8
	75 (25)	42.7 ± 3.7	62.0 ± 3.1	160.8 ± 14.3	130.0 ± 18.2	45.3 ± 5.3	59.0 ± 9.2
	100 (36)	41.3 ± 3.5	60.7 ± 7.3	170.0 ± 11.5	148.0 ± 4.6	43.1 ± 4.9	74.7 ± 13.2
	150 (50)	56.0 ± 11.1	60.0 ± 7.3	155.1 ± 8.8	155.6 ± 10.4	35.3 ± 1.6	68.0 ± 12.9
**S1h**	10 (10)	56.7 ± 6.8	64.0 ± 4.2	178.0 ± 13.1	138.0 ± 8.2	53.5 ± 3.6	59.3 ± 7.4
	50 (18)	53.3 ± 1.3	64.7 ± 8.1	153.3 ± 4.8	177.3 ± 7.4	59.3 ± 5.9	58.2 ± 3.4
	75 (25)	64.0 ± 7.7	70.2 ± 4.3	177.3 ± 12.7	145.5 ± 10.3	54.2 ± 4.3	59.5 ± 3.7
	100 (36)	64.0 ± 7.5	70.2 ± 6.7	168.3 ± 3.8	169.7 ± 17.8	50.7 ± 4.3	58.0 ± 5.0
	150 (50)	61.3 ± 5.8	68.0 ± 4.9	155.3 ± 14.4	146.7 ± 6.7	52.0 ± 9.2	58.5 ± 1.4
**Vehicle**		47.7 ± 2.5 ^a^	52.7 ± 2.7 ^a^	157.1 ± 8.1 ^a^	153.6 ± 17.1 ^a^	48.2 ± 3.2 ^a^	66.5 ± 3.9 ^a^
**Mutagen**		149.7 ± 10.6 ^b,^**	266.1 ± 25.1 ^c,^**	995.8 ± 85.9 ^d,^**	379.2 ± 23.5 ^c,^**	323.4 ± 31.2 ^e,^**	347.4 ± 16.4 ^f^^,^**

^a^ DMSO 50 µL; ^b^ 2-Nitrofluorene (2 μg∙plate^−1^); ^c^ 2-Aminoanthracene (1 μg∙plate^−1^); ^d^ Sodium azide (1 μg∙plate^−1^); ^e^ Methyl methane sulfonate (500 μg∙plate^−1^); ^f^ 2-Aminoanthracene (10 μg∙plate^−1^). ** *p* < 0.01 *vs.* vehicle (Anova + Dunnett’s multiple comparison post-test).

## 3. Experiment Section

### 3.1. Plant Material

Aerial parts of MS were collected in a wild area about 10 Km from Tarquinia city (Viterbo, Italy, latitude 42°20*'*23.3406*"*, longitude 11°46*'*16.3452*"*, Supplemental Material Figure S4) in three different days (7 July, 24 August and 28 September 2011). Since every year the MS field is completely ploughed, the age of the plants was less than one year. 

### 3.2. Essential Oil Extractions

Fresh aerial parts of the plant material (about 2.5 kg) were subjected to hydrodistillation using a 62 L steel distillator apparatus (Albrigi Luigi E0131, Verona, Italy) separating oils at interval time of 1, 2, 3, 6, 12 and 24 h. At each interval, the accumulated oil/water double phase (50 mL) was extracted three times with diethyl ether (20 mL). The organic layers were then dried on anhydrous Na_2_SO_4_, filtered and private of the solvent *in vacuo* to furnish pale yellow oils.

### 3.3. Essential Oil Analysis

*GC/MS conditions.* The gas chromatographic/mass spectrometric (GC/MS) analysis was carried out with a GC-MS and GC-FID by using a turbomass Clarus 500 GC-MS/GC-FID from Perkin Elmer instruments (Waltham, MA, USA). A Stabilwax fused-silica capillary column (Restek, Bellefonte, PA, USA) (60 m × 0.25 mm, 0.25 mm film thickness) was used with helium as carrier gas (1.0 mL∙min^−1^). GC oven temperature was kept at 60 °C for 5 min and programmed to 220 °C at a rate of 5 °C∙min^−1^, and kept constant at 220 °C for 30 min. Solvent delay 0–2 min. and scan time 0.2 s. MS was taken at 70 eV. Mass range was from 30 to 350 *m*/*z*. 1 μL of the extract was diluted in 1 mL of methanol and 1 μL of the solution was injected into the GC injector at the temperature of 280 °C. 

The main components of essential oils were identified at first by GC injection of analytical standard compounds comparing their retention times with those obtained in the essential oil analysis. A second confirmation was performed by GC-MS by comparison of the mass spectra with those characteristic of the standard sample and, if not commercially available, with the NIST and NBS Libraries (https://library.nist.gov/). Relative abundances of the separated components were derived by using the same instrumentation with the FID detector configuration.

### 3.4. Antimicrobial Assay 

The Minimum Inhibitory Concentration (MIC) was determined by micro-broth dilution method (microsterile plate) according to the Clinical and Laboratory Standards Institute/National Committee for Clinical Laboratory Standards (CLSI/NCCLS) Approved Standard M27-A3, 2008 [NCCLS]. The MIC was determined as the lowest concentration of drugs or essential oils at which no microbial growth was observed. Miconazole 0.5 mg∙mL^−1^ solution was prepared dissolving the agent in endotoxin free water. Solutions of essential oil (100 mg∙mL^−1^) were prepared in RPMI 1640. Briefly, to determine the MIC of TEOMS extracted at different times and in different months, or miconazole, RPMI-1640 supplemented with MOPS at pH 7 was used. TEOMS was diluted in RPMI-1640 supplemented with Tween 80 (final concentration of 0.001% *v*/*v*). The dilutions, 11 increasing concentrations, ranging from 0.012 to 12.48 mg∙mL^−1^ of the essential oil, were prepared in 96 well plates. The inoculum size was about 2.5 × 10^3^ cells∙mL^−1^. The plates were incubated at 30 °C for 24–48 h.

### 3.5. Mutagenicity Tests

*Chemicals and S9-based metabolic activation system.* All the substances used in the Ames test, including the mutagens 2-nitrofluorene (2NF) (CAS number 607-57-8; purity 98%), 2-aminoanthracene (2AA) (CAS number 613-13-8; purity 96%), sodium azide (SA) (CAS number 26628-22-8; purity > 99.5%), methyl methane sulfonate (MMS) (CAS number 66-27-3; purity 99%), and the media were obtained from Sigma-Aldrich Co (St. Louis, MO, USA). The TEOMS samples and the mutagens 2NF and 2AA were dissolved in DMSO (CAS number 67-68-5; purity > 99.5%), while SA and MMS in deionised water. 

S9 fraction (the liver post mitochondrial supernatant of rats treated with the mixture phenobarbital/β-naphthoflavone to induce the hepatic microsomal enzymes) was purchased from Moltox (Molecular Toxicology, Boone, NC, USA) and prepared just before use by adding: phosphate buffer (0.2 M) 500 µL, deionised water 130 µL, KCl (0.33 M) 100 µL, MgCl_2_ (0.1 M) 80 µL, S9 fraction 100 µL, glucose-6-phosphate (0.1 M) 50 µL and NADP (0.1 M) 40 µL. The final mixture was kept on ice during testing.

Bacterial strains. Salmonella ser. typhimurium TA98 (hisD3052gal biochl1008rfa1001 *uvr*BpKM101), *S. typhimurium* TA100 (hisG56galbiochl1005rfa1004 Δ*uvr*B pKM101) and Eschericia coli WP2*uvr*A (trpE65Δ*uvr*A) were kindly supplied by the Research Toxicological Centre (Pomezia, Rome, Italy). The genotypes of these strains were confirmed by testing the presence of specific genetic markers, in preliminary Strain Check Assays [41]; thus, the permanent cultures were prepared and frozen. The working cultures, used in each experiment, were obtained from the frozen permanent cultures after incubation overnight (16 h) at 37 °C to reach a concentration of approximately 1 × 10^9^ bacteria∙mL^−1^. In each experiment, the number of viable cells for each strain was defined according to Di Sotto *et al.* [41].

*Preliminary assays.* Preliminarily, the solubility in the final treatment mixture of three samples of *M. suaveolens* essential oil was assessed, in order to find the highest concentration to use in the following assays. Insolubility was defined as the formation of a precipitate in the final mixture evident to unaided eye. All the samples did not give any precipitate up to the highest concentration tested of 850 μg∙plate^−1^ (corresponding to 320 μg∙mL^−1^). Starting from this concentration, serial dilutions (dilution factor from 1:1.2 to 1:2.5) was prepared and tested in the cytotoxicity assay. Cytotoxic effects were evaluated as a reduction (>70%) in the number of revertant colonies and as a change in the auxotrophic background growth (background lawn), in comparison with the control plates [41]. To perform the test, solutions of test substances (50 µL) were added to an overnight culture (100 µL) and S9 mixture or phosphate buffer (0.1 M; 500 µL). The mixture was pre-incubated under shaking at 37 °C for 30 min, then it was added with top agar (2 mL) containing 10% of histidine/biotin (0.5 mM) for TA98 and TA100, and 10% of tryptophan (0.5 mM) for WP2*uvr*A; finally it was poured onto a minimal agar plate. After incubation at 37 °C for 48 h, the plates were examined, the background lawn was observed and the bacterial colonies were scored. 

*Mutagenicity assays.* Mutagenicity of the substances was assayed using the pre-incubation method [41]. Different dilutions (dilution factor from 1:1.2 to 1:1.5) of the three samples of OEMS were prepared starting from the highest non-toxic concentration. Therefore, their effect on the spontaneous revertant colonies of TA98, TA100 and WP2*uvr*A strains was tested in comparison with the vehicle (DMSO, 2% v/v; negative control). In each experiment, some plates treated with known mutagens (positive controls) were also included, in order to verify the ability of the experimental system to detect a genotoxic effect. As mutagens we used 2-nitrofluorene (2NF; 2 μg∙plate^−1^ for TA98), sodium azide (SA; 1 μg∙plate^−1^ for TA100) and methyl methanesulfonate (MMS; 500 μg∙plate^−1^ for WP2*uvr*A), in the absence of the metabolic activator S9, while 2-aminoanthracene (2AA; 1 μg∙plate^−1^ for TA98 and TA100 and 10 μg∙plate^−1^ for WP2*uvr*A) in the presence of S9. These concentrations of mutagens, obtained from the linear part of the concentration-response curve, were chosen as they increased the number of revertant colonies at least two-fold above the control value. The experiments were repeated at least twice and each concentration was tested in triplicate. To perform the test, an overnight culture (100 µL) was added to the solutions of the samples tested (50 µL) plus S9 mixture or phosphate buffer (0.1 M) (500 µL). The mixture was gently vortexed in a sterile tube then was incubated under shaking at 37 °C for 30 min. After the pre-incubation, the tubes were added with top agar (2 mL) containing 10% of histidine/biotin (0.5 mM) for TA98 and TA100, and 10% of tryptophan (0.5 mM) for WP2*uvr*A, then were gently vortexed and poured onto a minimal agar plate. The plates were incubated at 37 °C for 72 h and then examined. The histidine- or tryptophan-independent revertant colonies and the viable cells were scored and the bacterial background lawn was observed. A positive response in the test was defined as an increase (at least two-fold above the control) in histidine- or tryptophan-independent revertant colonies in each strain [41].

*Statistical analysis.* All values are expressed as mean ± SE. The one-way analysis of variance (one-way ANOVA), followed by Dunnett’s multiple comparison post-test, was used to verify the significance of a positive response. A *p* value < 0.05 was considered statistically significant. Statistical analysis was performed with GraphPad Prism™ (Version 4.00) software (GraphPad Software, Inc., San Diego, CA, USA).

## 4. Conclusions

In recent years, the interest in essential oils has continually increased; nevertheless, no detailed study on the extraction procedure has yet been reported considering both extraction time and period. In line with our previous studies on TEOMS, a systematic extraction procedure was applied to three different periods during the summer of 2011, and at various extraction times. In order to monitor the biological variability, the essential oils were assayed by means of a classical antimicrobial protocol that suggested piperitenone oxide as the principal active chemical constituent responsible for the antifungal activity. Although this was somewhat an expected result, the herein systematic procedure revealed that other constituents may participate actively in the biological effect. In particular, the extract **S24h**, although lacking piperitenone oxide, revealed a single digit micromolar activity (pMIC = 5.92, Supplemental Material Table S1) that can be mainly ascribed to the whole mixture where some of them act in a synergistic way and some act uncooperatively; as a matter of fact, sample **A2h** is five times more effective than sample **J1h** which contains less PO (compare their compositions in Table 2, Table 3 and Table 5). It could be speculated that for **A2h**, α-cadinol and α-cubebene (the more abundant components after PO) could have some positive and additive biological role; on the other hand, for **J1h** it could be that α-pharnesene (the most abundant after PO) could demonstrate some anti-synergism with PO. The lack of mutagenic effect in the tested essential oils somewhat supports the safe use of *Mentha suaveolens* essential oil as a food additive. In conclusion, from data herein presented, the answers to the questions in the introduction can be partially answered. In relation to the biological activity, the period of extraction seems to be of marginal importance as any essential oil collected in the first three hours showed similar anti-candida activity and was deprived of any mutagenicity effects. On the other hand, consideration should also be given to the EO production yield or the chemical composition. Harvesting in August lead to the higher EO production, while in July the higher piperitenone oxide percentage was obtained. Regarding the second question (How long should the hydrodistillation time be?), the answer depends on what the study is conducted for. To obtain EOs with the highest PO content, hydrodistillation of 1 to 3 must be undertaken, while for extraction of different components, like *p*-cymenene, cinerolone and demelverine, 24 h of hydrodistillation should be carried out.

## Supplementary Materials

Supplementary materials can be accessed at: http://www.mdpi.com/1420-3049/20/06/9640/s1.
