# Supplementary material for: Multidisciplinary Approach to Determine the Optimal Time and Period for Extracting the Essential Oil from Mentha suaveolens Ehrh
Source: Molecules. 2015 May 26;20(6):9640–55. doi: 10.3390/molecules20069640 (PMC6272612; doi:10.3390/molecules20069640)
Supplement: Supplementary file 1 [file molecules-20-09640-s001.pdf]

Supplementary Materials

Table S1. Anti-*Candida albicans* activities of the 18 TEOMS extracts.

Sample [1]	Weighted MW [2]	MIC mg/mL	MIC (µmol/L)	pMIC (mol/L) [3]	PO %
**J1h**	166.67	0.10	0.58	6.24	87.25
**J2h**	168.06	0.10	0.58	6.24	70.59
**J3h**	170.89	0.10	0.57	6.25	65.56
**J6h**	186.44	6.25	33.52	4.47	26.03
**J12h**	190.09	6.25	32.88	4.48	14.78
**J24h**	193.27	12.50	64.68	4.19	-
**A1h**	177.24	0.10	0.55	6.26	65.05
**A2h**	171.98	0.02	0.14	6.86	77.51
**A3h**	176.85	0.10	0.55	6.26	50.01
**A6h**	182.34	0.78	4.28	5.37	16.90
**A12h**	180.01	3.12	17.33	4.76	2.43
**A24h**	179.64	6.25	34.79	4.46	-
**S1h**	175.85	0.20	1.11	5.96	38.69
**S2h**	175.05	0.20	1.11	5.95	35.64
**S3h**	165.42	0.10	0.59	6.23	69.52
**S6h**	182.69	6.25	34.21	4.47	13.20
**S12h**	182.35	6.25	34.28	4.47	5.53
**S24h**	180.18	0.20	1.08	5.97	5.61
**Miconazole**	479.140	0.016	0.033	7.48	

[1] Samples names were obtained by merging the month first letter and extraction time as reported in Table 1.
[2] Weighted MWs were calculated through a weighted sum of the chemical constituents' MW multiplied by their relative percentages. [3] pMIC = −log10(MIC expressed in molar).

Table S2. Chemical structures, names, MW and CAS Number of all 48 chemical constituents found in the 18 essential oils.

# [1]	Chemical Structure and Name	MW	CAS	# [1]	Chemical Structure and Name	MW	CAS
**1**	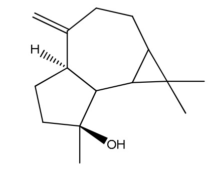 (−)-spathulenol	220.35	77171-55-2	25	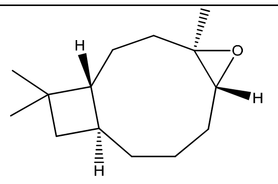 β-caryophyllene oxide	220.35	1139-30-6
**2**	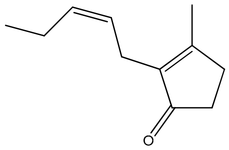 (z)-jasmone	164.25	488-10-8	26	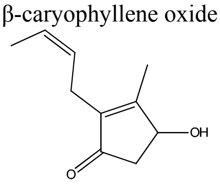 cinerolone	166.22	17190-74-8
**3**	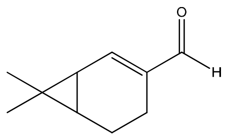 2-caren-10-al	150.22	Not available	27	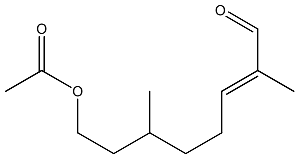 citronellyl acetate	198.31	150-84-5

Table S2. *Cont.*

# [1]	Chemical Structure and Name	MW	CAS	# [1]	Chemical Structure and Name	MW	CAS
4	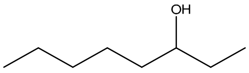 3-octanol	130.23	589-98-0	28	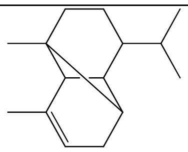 copaene	204.36	3856-25-5
5	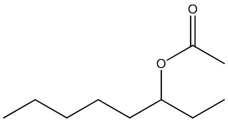 3-octanol acetate	172.27	4864-61-3	29	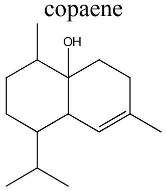 cubenol	222.37	21284-22-0
6	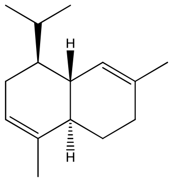 α-cadinene	204.36	24406-05-1	30	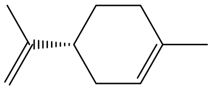 d-limonene	136.23	5989-27-5
7	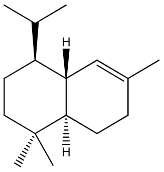 α-cadinol	222.37	481-34-5	31	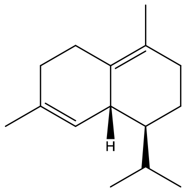 delta-cadinene	204.36	483-76-1
8	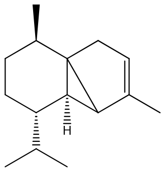 α-cubebene	204.36	17699-14-8	32	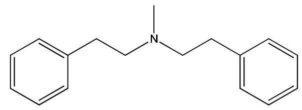 demelverine x	239.36	13977-33-8
9	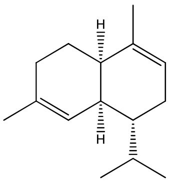 α-muurolene	204.35	31983-22-9	33	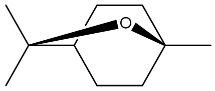 eucalyptol	154.25	470-82-6
10	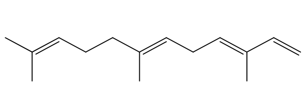 α -farnesene	204.35	502-61-4	34	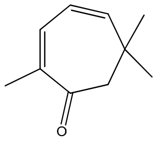 eucarvone	150.22	503-93-5
11	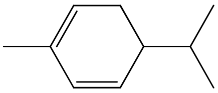 α-phellandrene	136.23	99-83-2	35	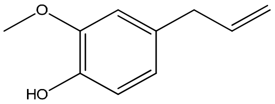 eugenol	164.20	97-53-0

Table S2. *Cont.*

# [1]	Chemical Structure and Name	MW	CAS	# [1]	Chemical Structure and Name	MW	CAS
**12**	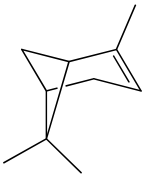 α-pinene	136.23	80-56-8	36	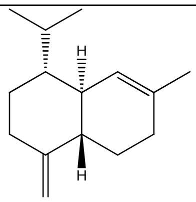 gamma-cadinene	204.36	1460-97-5
**13**	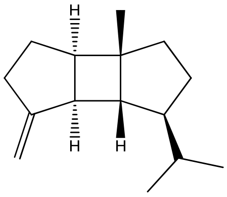 β-bourbonene	204.35	5208-59-3	37	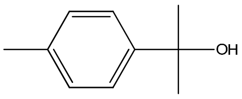 p-cymen-8-ol	150.22	1197-01-9
**14**	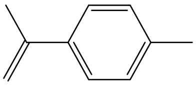 p-cymenene	132.20	1195.32.0	38	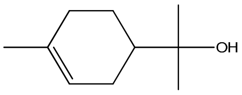 α-terpineol	154.25	98-55-5
**15**	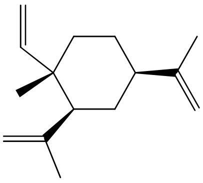 β-elemene	204.35	515-13-9	39	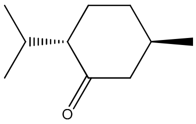 p-menthone	154.25	21060-23-1
**16**	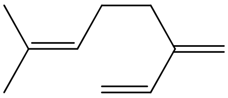 β-myrcene	136.23	123-35-3	40	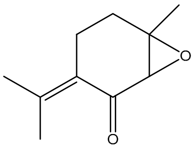 piperitenone oxide	166.22	35178-55-3
**17**	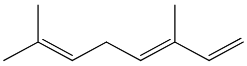 β-ocymene	136.23	13877-91-3	41	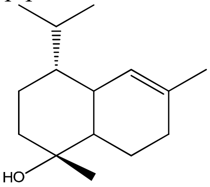 tau-cadinol	222.37	1474790
**18**	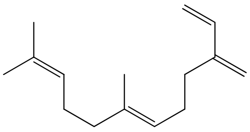 β-farnesene	204.35	18794-84-8	42	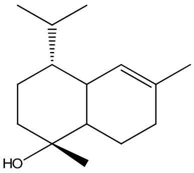 tau-muurolol	222.37	19912-62-0
**19**	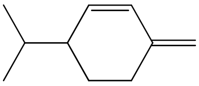 β-phellandrene	136.24	555-10-2	43	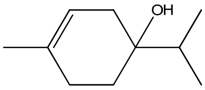 terpinen-4-ol	154.25	562-74-3

Table S2. *Cont.*

# [1]	Chemical Structure and Name	MW	CAS	# [1]	Chemical Structure and Name	MW	CAS
**20**	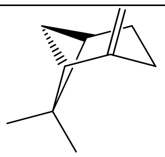 β –pinene	136.23	18172-67-3	44	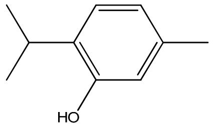 thymol	150.22	89-83-8
**21**	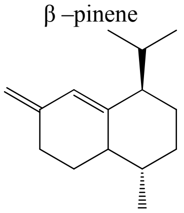 bicyclosesquiphellandrene	204.35	54324-03-7	45	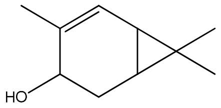 trans-2-caren-4-ol	152.23	4017-82-7
**22**	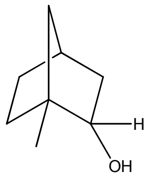 borneol	154.25	507-70-0	46	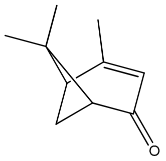 verbenone	150.21	80-57-9
**23**	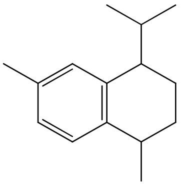 Calamenene	202.34	483-77-2	47	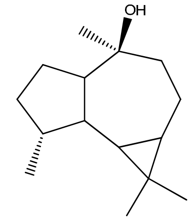 veridiflorol	222.37	552-02-3
**24**	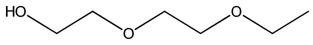 Carbitol	134.20	111-90-0	48	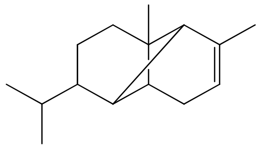 ylangene	204.35	14912-44-8

[1] # indicate the compound identification number.

Table S3. Chemical composition of TEOMS extracted in July, August and September. Values are in % weight rounded to the second decimal place.

# [1]	Name	Sample [2]																	
		**J1h**	**J2h**	**J3h**	**J6h**	**J12h**	**J24h**	**A1h**	**A2h**	**A3h**	**A6h**	**A12h**	**A24h**	**S1h**	**S2h**	**S3h**	**S6h**	**S12h**	**S24h**
**1**	(−)-spathulenol	2.17	8.18	8.99	14.23		18.35	0.79	1.48	2.47	2.12	0.32		3.67	8.95	2.54	3.62	1.69	0.99
**2**	(z)-jasmone																		0.54
**3**	2-caren-10-al																	0.32	0.76
**4**	3-octanol	0.18						0.73			0.11	0.07			3.13	4.74	0.82	0.61	1.19
**5**	3-octanol acetate	0.11						2.08	1.02	0.9					4.9	2.2	0.15	0.12	0.15
**6**	α-cadinene		0.24																
**7**	α-cadinol		0.5	1.75	9.09	10.69	7.62				0.78	0.48	0.09	0.34		0.49	1.73	1.22	0.44
**8**	α-cubebene	0.3	0.31		0.09			5.07	4.36	10.08	3.64	0.45	0.14			0.23	1.97	0.96	1.7
**9**	α-muurolene													0.09		0.13	0.51	0.58	0.15
**10**	α-pharnesene							5.15	8.01	16.54	9.09	2.56	0.37	1.6	10.15	0.11	12.09	6.42	6.31
**11**	α-phellandrene							0.11											
**12**	α-pinene							1.84											0.83
**13**	β-bourbobene	0.17																	
**14**	p-cymenene							0.87	0.33	2.54	7.74	18.24	35.22		0.83		1.09	5.04	26.64
**15**	β-elemene										0.38	0.07							
**16**	β-myrcene							1.42											0.35
**17**	β-ocymene							0.59											0.25
**18**	β-pharnesene							0.73	1.03	2.29	2.24	0.89	0.18	0.3	1.32	0.11	5.46	3.23	0.48
**19**	β-phellandrene							0.42											0.1
**20**	β-pinene							0.68											
**21**	bicyclosesquiphellandrene							0.88	0.98	2.37	1.89	0.41				0.19	2.33	1.16	0.77
**22**	borneol	0.12						0.28	0.29	0.39	0.37	0.3	0.2				0.23	0.34	0.14
**23**	calamenene	0.58	2.09	2.52	3.75	10.63	4.32	0.44	1.07	1.56	1.33	0.65		1.68	3.01	1.26	1.87	1.21	0.8
**24**	carbitol				0.55		2.36												
**25**	β-caryophyllene oxide	5.32	13.67	12.65	14.37	5.27	9.24	0.5	0.32	1.04	8.3	17.25	12.93	14.16	14.08	6.3	5.77	9.65	6.37
**26**	cinerolone	0.17	0.09				7.71				23.12	34.49	38.79	18.82			2.66	13.04	18.96
**27**	citronellylacetate				0.63	3.21	5.45												

Table S3. *Cont.*

# [1]	Name	Sample [2]																	
		**J1h**	**J2h**	**J3h**	**J6h**	**J12h**	**J24h**	**A1h**	**A2h**	**A3h**	**A6h**	**A12h**	**A24h**	**S1h**	**S2h**	**S3h**	**S6h**	**S12h**	**S24h**
**28**	copaene				0.06		0.76				0.79	0.47		0.18	0.21	0.31	1.55	1.67	0.49
**29**	cubenol	0.2		1.92	7.46	6.17	4.24				0.36	0.09	0.04	0.4	1.77	0.7	1.44	0.84	0.18
**30**	d-limonene							6.22							0.69				4.84
**31**	δ-cadinene				0.12		4.89	0.09	0.27	1.07	2.32	1.54	0.44	0.89		0.14	3.05	3.33	1.25
**32**	demelverine	0.59	0.51	2.18	9.52	43.46	20.28	0.13	1.1	3.14	7.46	8.82	5.9	6.23	0.68	1.84	2.46	4.9	5.22
**33**	eucalyptol							4.21	0.47						1.16	0.43			2.24
**34**	eucarvone	0.08																	
**35**	eugenol													1.42			2.07	3.16	0.55
**36**	gamma-cadinene						0.86				0.51	0.26	0.06						
**37**	p-cymen-8-ol	0.12						0.21	0.54	0.66	0.94	0.97	0.55	1.67	9.22	0.37	26.77	24.68	4.52
**38**	p-menth-1-en-8-ol	0.14													0.55	0.2	0.88	0.72	0.22
**39**	p-menthone							0.39	0.46	2.63	5.7	5.61	2.25			4.46	1.21	1.57	3.61
**40**	piperitenone oxide	87.25	70.59	65.56	26.03	14		65.05	77.51	50.01	16.9	2.43		38.69	35.64	69.52	13.2	5.53	5.61
**41**	tau-cadinol			0.72	1.39		1.37												
**42**	tau-muurolol		0.18	1.23	2.14		3.29												
**43**	terpinen-4-ol															0.16	0.3		0.16
**44**	thymol										0.39	0.32	0.24	1.34			0.25	1.04	0.15
**45**	trans-2-caren-4-ol																0.2		
**46**	verbenone	1.29	1.15		2.98	6.56	6.43	0.27	0.31	0.81	2.47	3.14	2.58	8.11	1.98	2.3	3.68	5.48	2.28
**47**	veridiflorol	1.21	2.48	2.49	7.59		2.83				0.39	0.17		0.42	1.74	0.99	1.8	0.96	0.28
**48**	ylangene							0.85	0.43	1.5	0.64						0.83	0.31	0.46

[1] # indicate the compound identification number; [2] Samples names were obtained by merging the month first letter and extraction time as reported in Table 1.

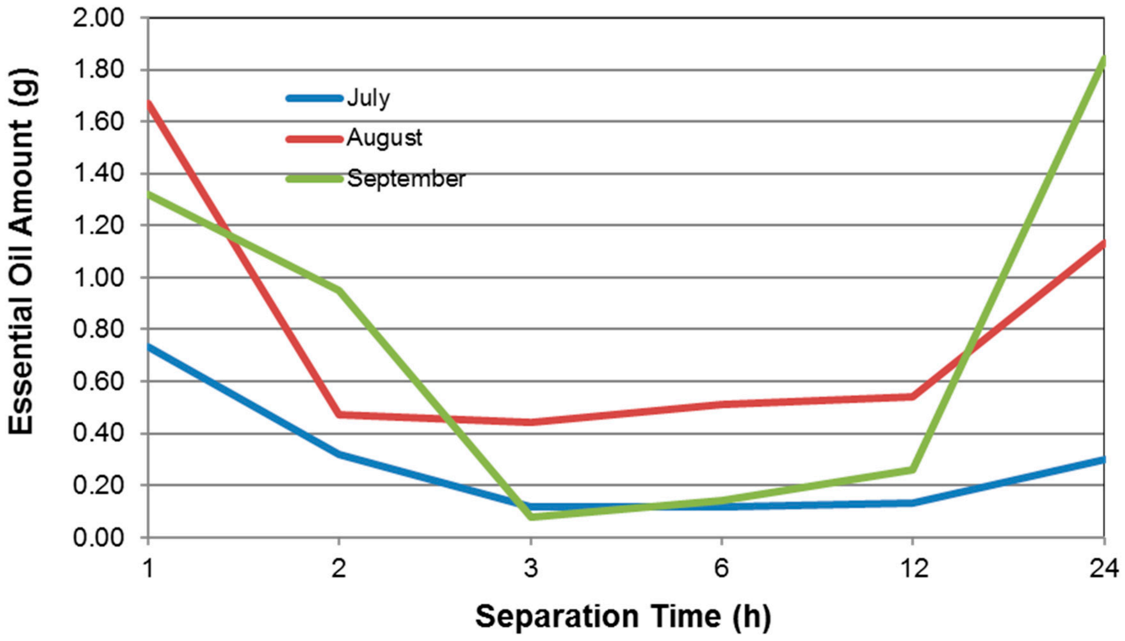

Figure S1. Fraction of essential oil amount during extraction at different times.

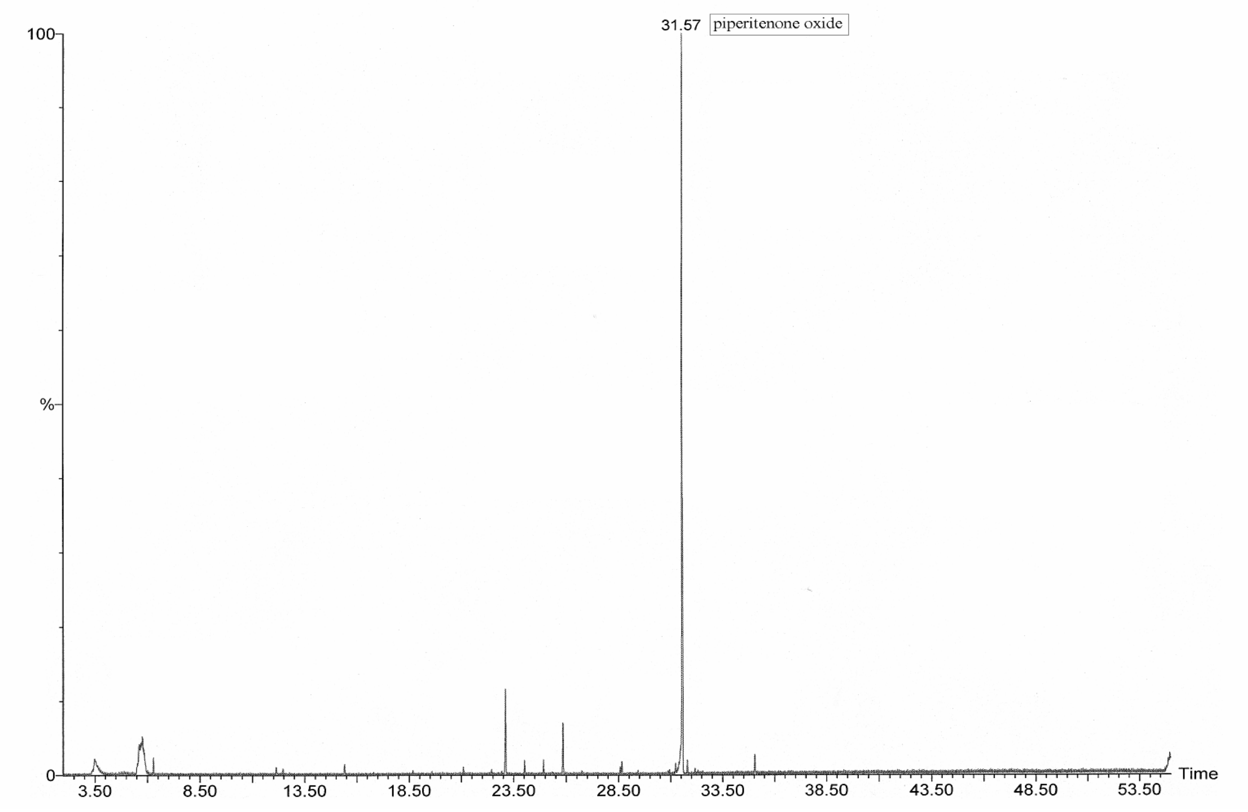

(A)

Figure S2. *Cont.*

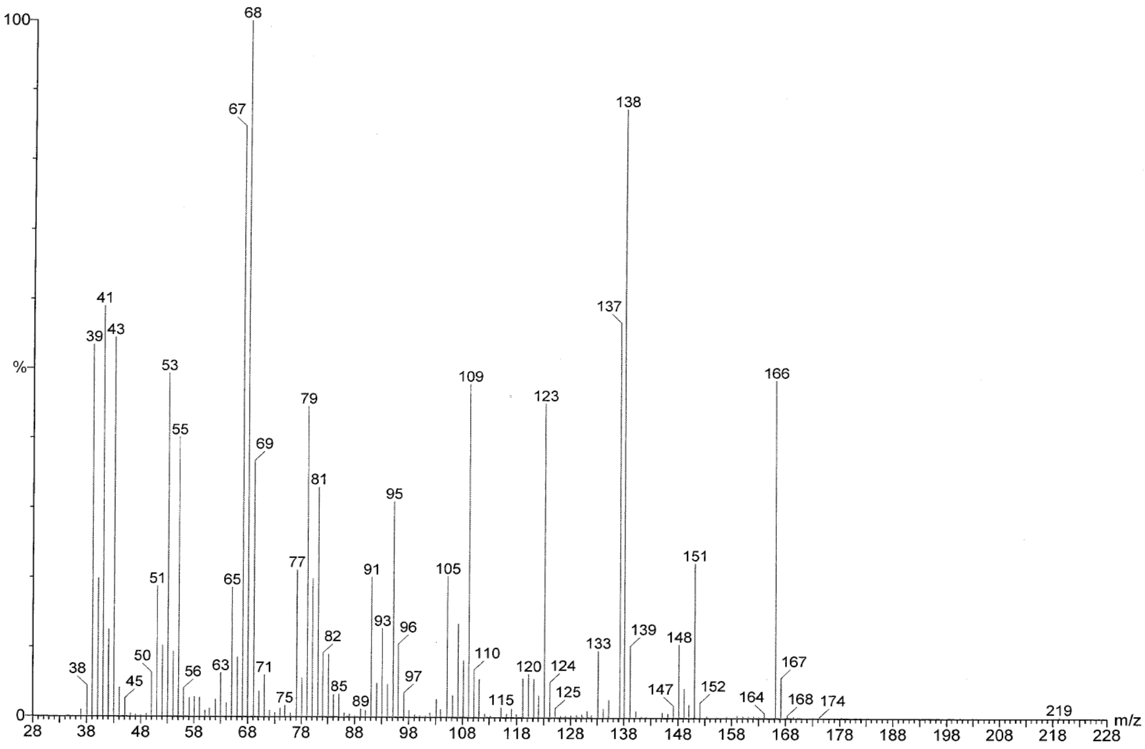

(B)

Figure S2. (**A**) Chromatogram of extract **A2h** showing piperitenone oxide as the main component; (**B**) Mass spectrum of the peak at retention time of 31.57 min. corresponding to piperitenone oxide.

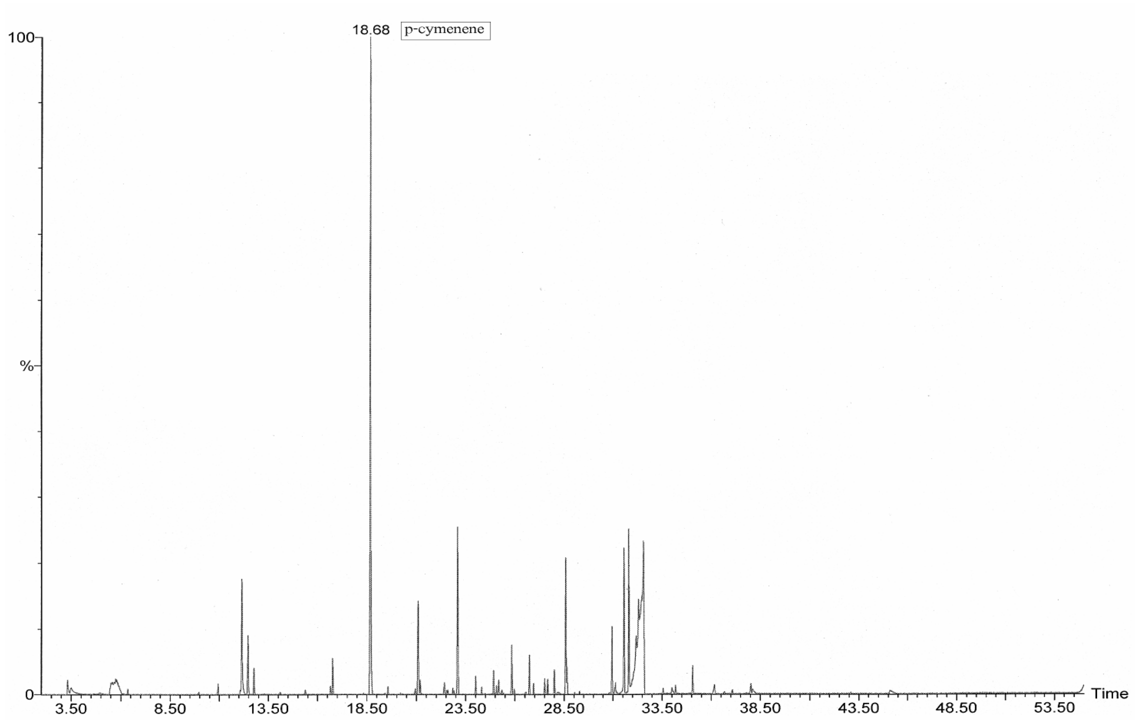

(A)

Figure S3. *Cont.*

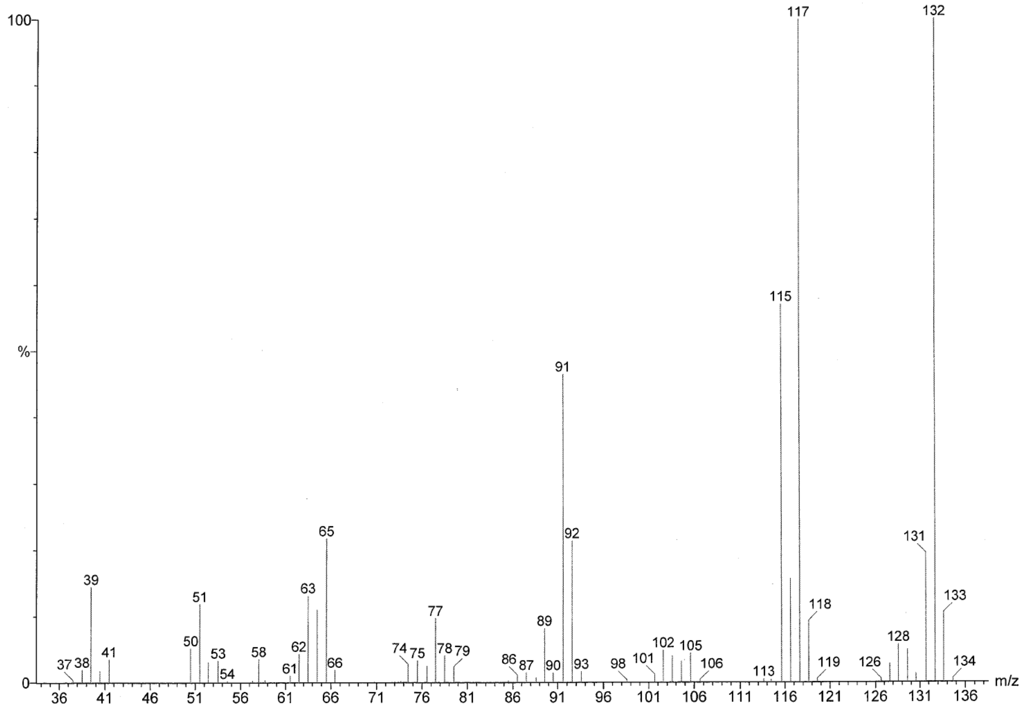

(B)

Figure S3. (**A**) Chromatogram of extract **S24h** showing p-cymenene as the main component; (**B**) Mass spectrum of the peak at retention time of 18.68 min. corresponding to p-cymenene.

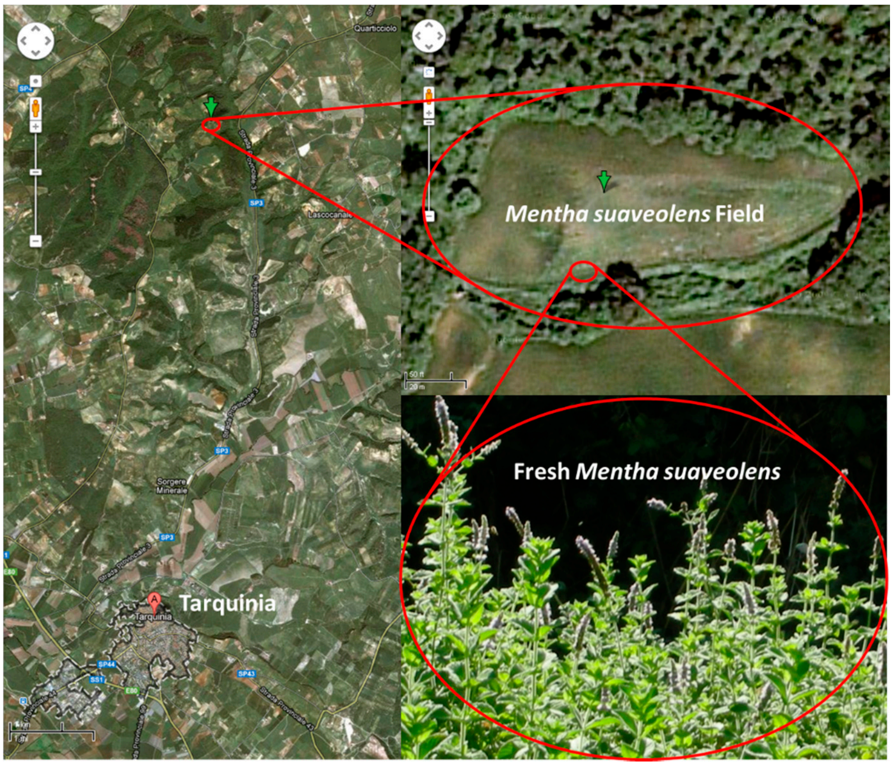

Figure S4. Left: Satellite view of the Tarquina's area where plant material was collected. **Right above**: zoom on the field were wild *M. suaveolens* was found at world coordinates latitude 42°20′23.3406″, longitude 11°46′16.3452″. **Right bottom**: picture of fresh plant in the field.
